# Chest Pain After Vomiting: Recognizing Boerhaave Syndrome in the Emergency Department

**DOI:** 10.7759/cureus.97219

**Published:** 2025-11-19

**Authors:** Mahnoor Tayyib, Muhammad Faizan Latif, Ahmed Sakr, Amr Mahmoud

**Affiliations:** 1 Emergency Medicine, Lincoln County Hospital, United Lincolnshire Hospitals NHS Trust, Lincoln, GBR; 2 Acute Medicine, Lincoln County Hospital, United Lincolnshire Hospitals NHS Trust, Lincoln, GBR; 3 Emergency Department, Lincoln County Hospital, United Lincolnshire Hospitals NHS Trust, Lincoln, GBR

**Keywords:** boerhaave’s syndrome, chest pain, oesophageal rupture, surgical emphysema, vomiting

## Abstract

Boerhaave syndrome is a rare but potentially fatal condition characterized by spontaneous transmural rupture of the esophagus, most often triggered by a sudden rise in intraluminal pressure against a closed glottis following forceful retching. Its clinical presentation can mimic other life‑threatening thoracic emergencies such as myocardial infarction, pulmonary embolism, or aortic dissection. A 50-year-old man presented to the Emergency Department with sudden onset of severe central chest pain radiating to the left side associated with shortness of breath, following multiple episodes of forceful vomiting. On examination, he was hemodynamically stable. Chest and soft-tissue neck radiographs demonstrated surgical emphysema, and a contrast-enhanced CT scan of the chest confirmed esophageal rupture with extraluminal air and food residue. The patient was managed with intravenous fluids, broad-spectrum antibiotics, and urgent cardiothoracic referral for definitive management. This case highlights the importance of maintaining a high index of suspicion for Boerhaave syndrome in patients presenting with chest pain following episodes of forceful vomiting, as early recognition and prompt intervention are vital to reduce morbidity and mortality.

## Introduction

Boerhaave syndrome is an uncommon but life-threatening gastrointestinal emergency resulting from spontaneous transmural perforation of the esophagus after an abrupt increase in intraluminal pressure against a closed upper esophageal sphincter [1]. It accounts for approximately 10% to 15% of all esophageal perforations and has an estimated incidence of 3.1 per million per year. Delayed diagnosis contributes to mortality rates that may exceed 40%, whereas survival improves to over 80% to 90% when intervention occurs within the first 24 hours.

The classic Mackler triad (vomiting, chest pain, and subcutaneous emphysema) is present in only about 14% of cases, making clinical identification challenging [1,2]. Risk factors include heavy alcohol consumption, overeating, prior esophageal disease, and activities causing sudden Valsalva-type pressure surges. Understanding the underlying pathophysiology and maintaining vigilance in at-risk patients are essential for early diagnosis [3].

Contrast-enhanced computed tomography (CT) is highly sensitive (92-100%) for detecting esophageal wall thickening, pleural effusion, pneumothorax, and hydrothorax [3]. Early recognition and treatment are critical, significantly reducing mortality to below 10%. While surgical repair remains the standard approach in most presentations of Boerhaave syndrome, this case is distinctive because the patient was successfully managed conservatively. This highlights the importance of individualized assessment, careful patient selection, and the growing recognition that non-operative strategies may be appropriate in select clinically stable cases.

In conclusion, Boerhaave syndrome is a rare esophageal disease, with a great risk of complications and death. It is known for a high mortality rate of up to 40%. Timely suspicion and diagnosis, together with individualized treatment, improve the prognosis [4,5].

## Case presentation

A 50‑year‑old man with no known comorbidities presented to the Emergency Department with acute, severe central chest pain radiating to the left side. The pain began immediately after a cluster of forceful vomiting episodes lasting approximately 10 to 15 minutes, triggered after consumption of pork meat. He reported progressive shortness of breath and a persistent sensation of obstruction in his chest. He denied alcohol intake, smoking, or prior esophageal pathology.

The patient arrived in the Emergency Department one hour after onset of pain and was alert and oriented. Vital signs were as follows: blood pressure 159/76 mmHg, heart rate 95 beats/min, respiratory rate 22 breaths/min, and oxygen saturation 97% on room air. Cardiovascular examination was normal. Respiratory examination revealed palpable crepitus extending across the upper chest and into the neck region, consistent with surgical emphysema. Breath sounds were clear bilaterally. The abdomen was soft and non‑tender.

Laboratory investigations including complete blood count, renal and liver function tests, serum electrolytes, and troponin were within normal limits. Given the temporal association with vomiting and the detection of subcutaneous emphysema, esophageal perforation was strongly suspected.

**Figure 1:**
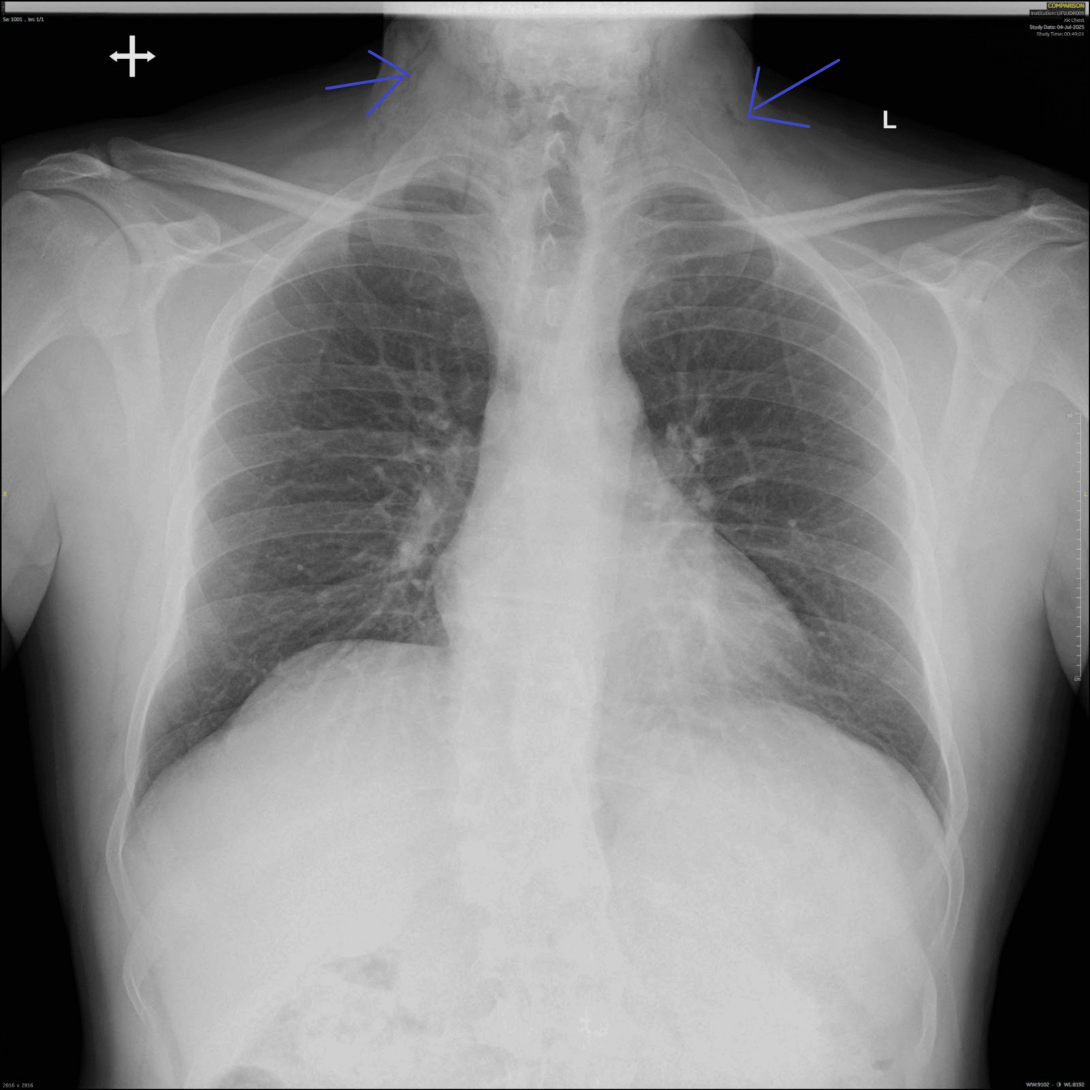
Posteroanterior chest x-ray/lateral soft tissue neck view Extensive surgical emphysema noted within the upper mediastinum along with soft tissues of the neck. No evidence of retained foreign body within the prevertebral soft tissues. Appearances raise the suspicion of an oesophageal perforation.

**Figure 2:**
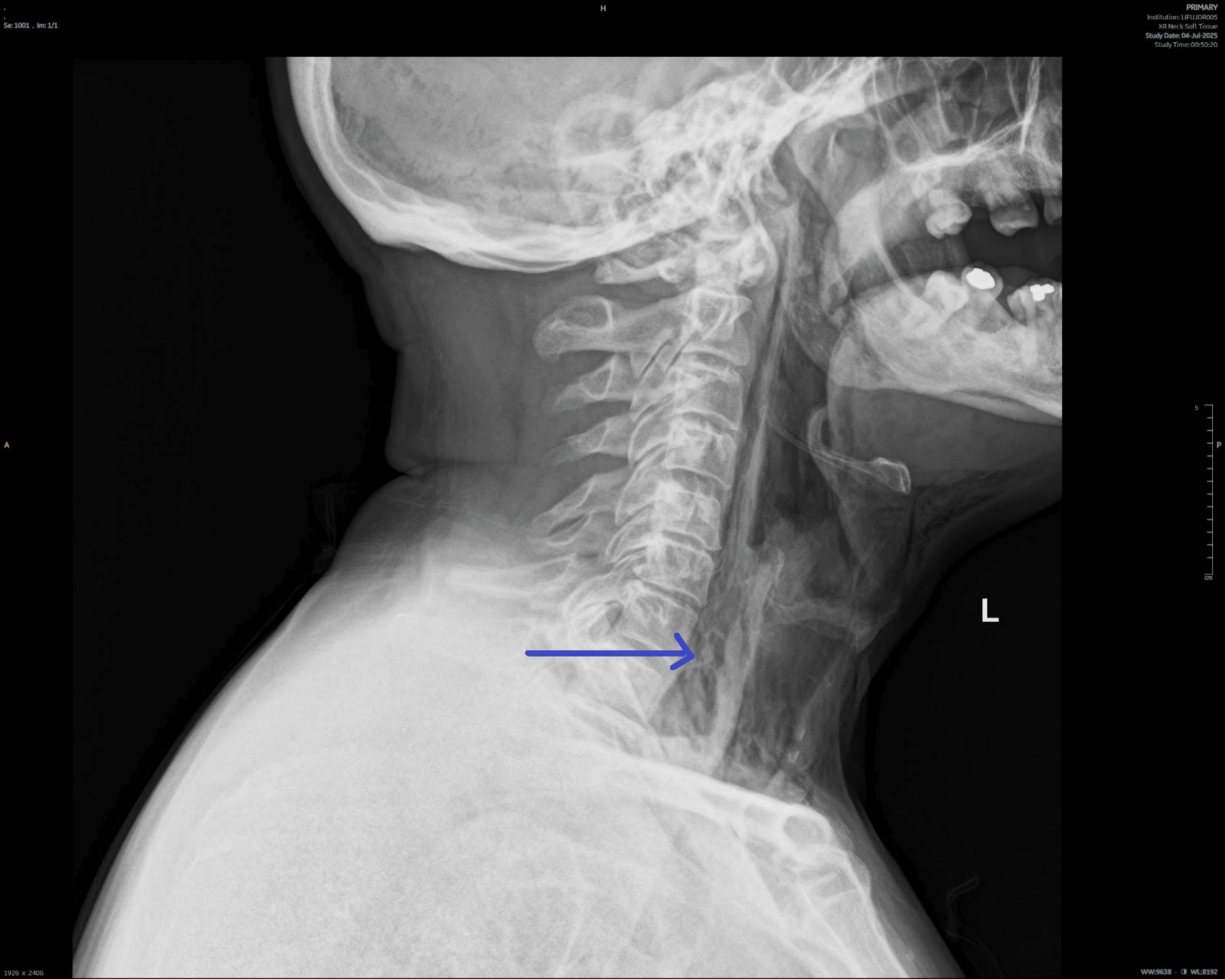
CT of the neck with contrast Pneumomediastinum noted mainly posteriorly along with soft tissue neck emphysema. Appearances are compatible with esophageal rupture.

**Figure 3:**
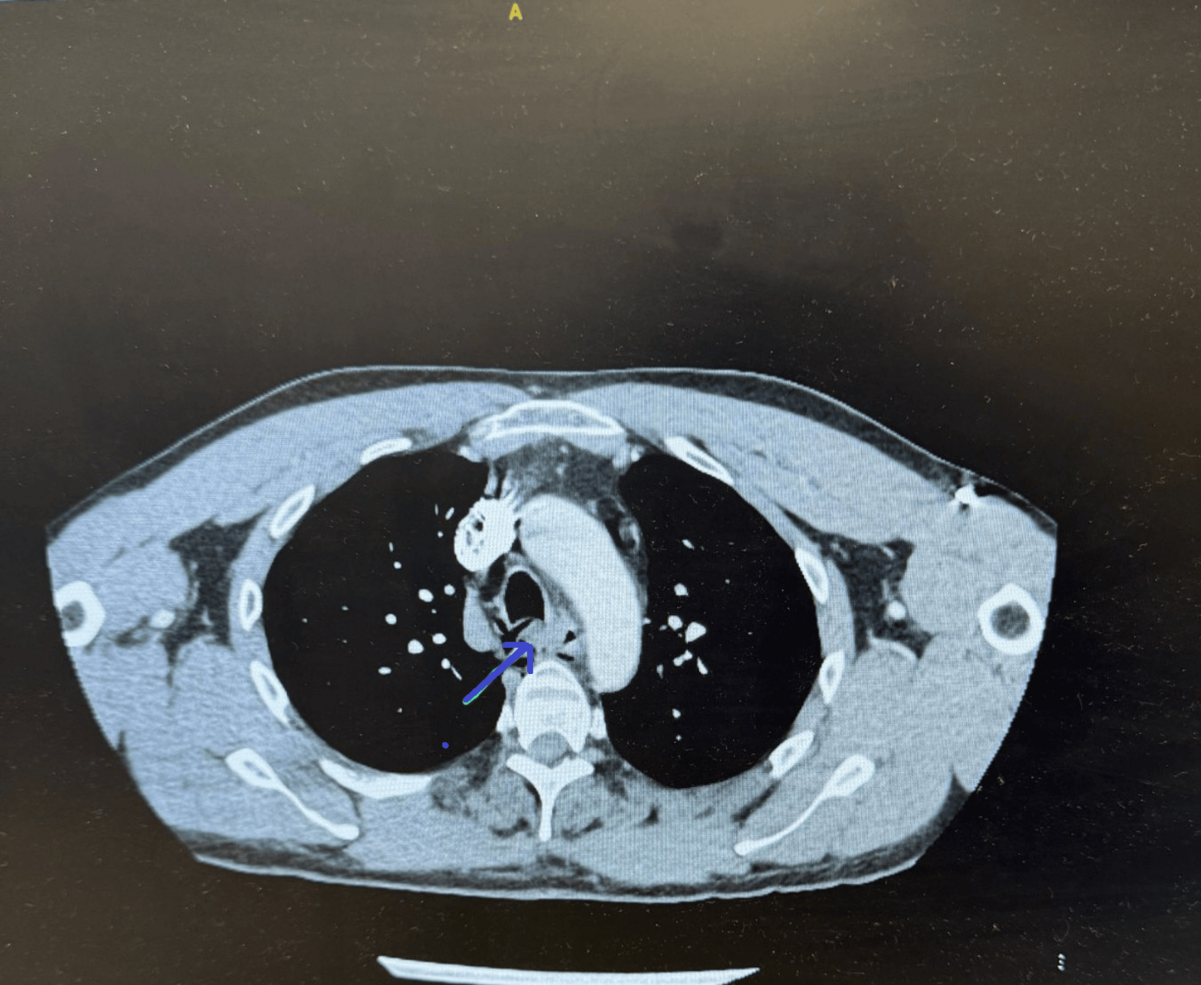
CT of the neck with contrast showing esophageal blockade with rupture

**Figure 4:**
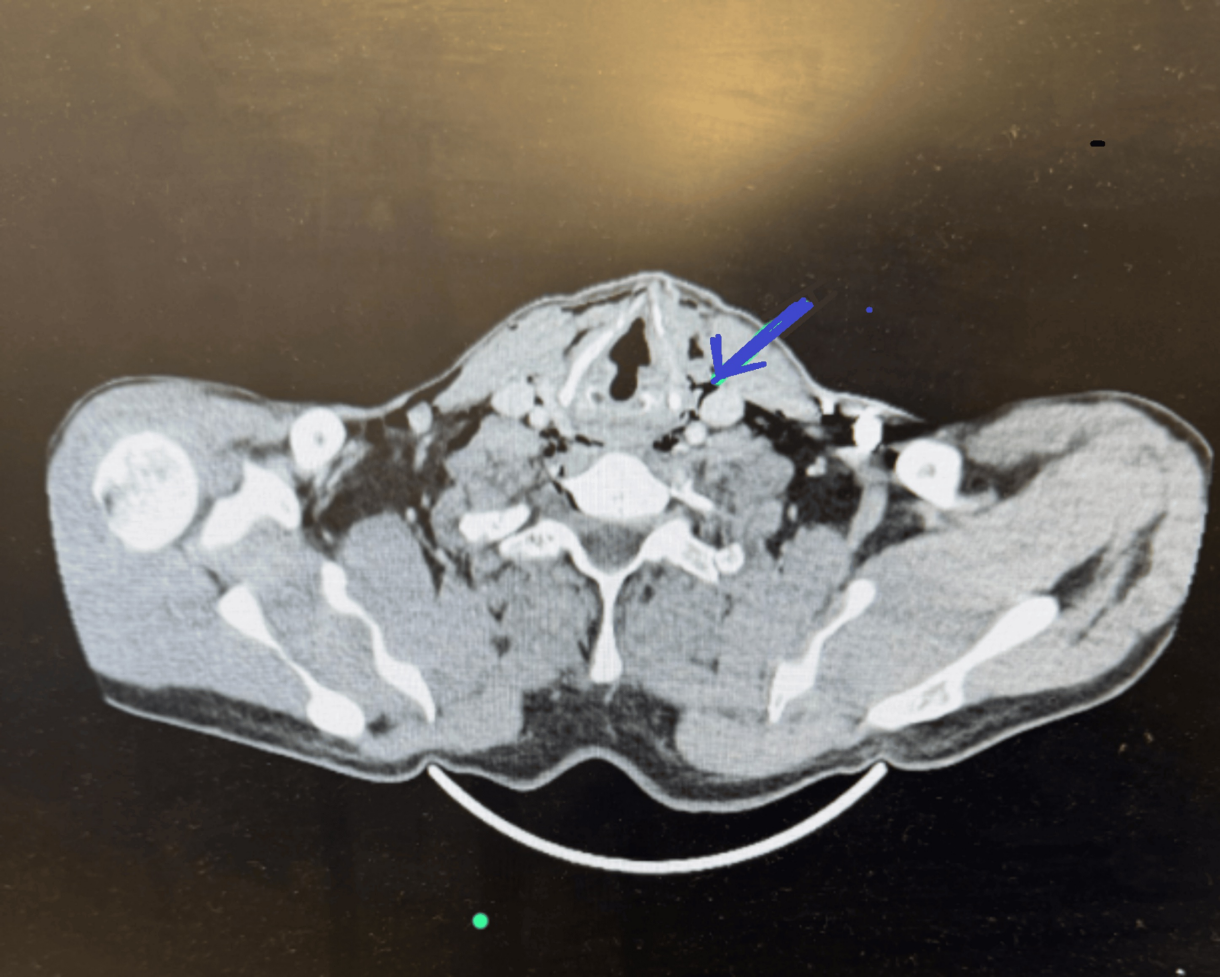
CT of the neck with contrast showing emphysema due to rupture

**Figure 5:**
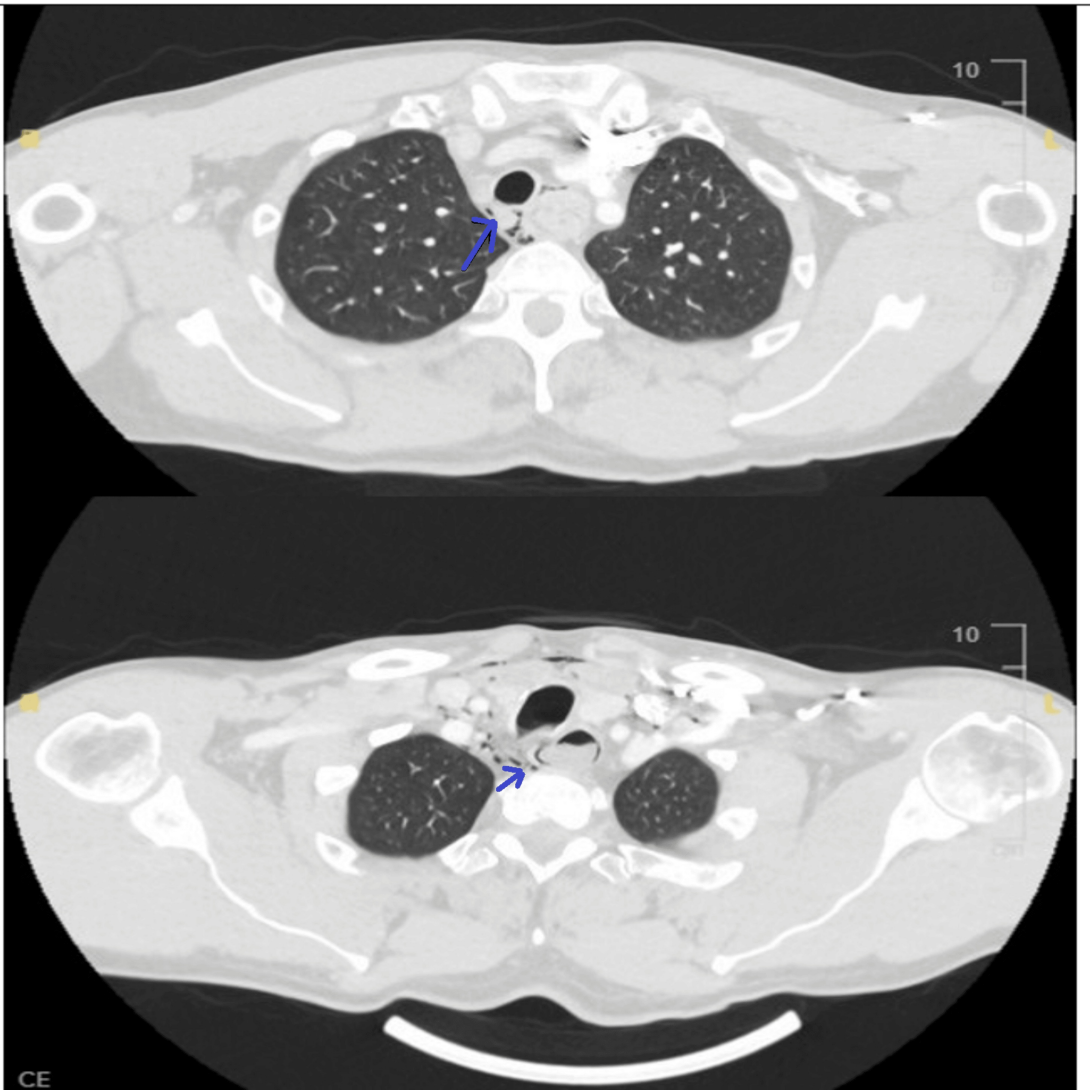
CT of the neck and thorax with contrast Pneumomediastinum noted mainly posteriorly along with soft tissue neck emphysema. Appearances are compatible with esophageal rupture.

A chest radiograph (Figure 1) demonstrated evidence of subcutaneous emphysema and possible mediastinal air. Subsequent CT of the neck and thorax with contrast (Figures 2-5) was performed for further evaluation and confirmation of the suspected diagnosis.

The patient received intravenous fluids and broad-spectrum antibiotics in the Emergency Department. A surgical referral was made immediately after CT confirmation, and he was transferred to a tertiary cardiothoracic center within two hours of imaging. He was commenced on oxygen therapy, blood cultures were obtained, and empirical antibiotics were continued. The patient remained hemodynamically stable and was managed conservatively. He was discharged on day 20 with advice to incorporate small, frequent meals, and remained asymptomatic at his two-month follow-up.

## Discussion

Boerhaave syndrome is a spontaneous, transmural esophageal rupture after a sharp intraluminal pressure surge against a closed cricopharyngeus. It is rare but highly lethal without rapid recognition and source control. The classic Mackler's triad (vomiting, chest pain, subcutaneous emphysema) is often incomplete; thus, clinicians must rely on a high index of suspicion plus careful examination for surgical emphysema. Crepitus in the neck or upper chest can be the decisive bedside clue that escalates imaging. The condition mimics other causes of acute chest pain such as myocardial infarction, pulmonary embolism, and aortic dissection. Distinguishing features include recent vomiting and the presence of subcutaneous emphysema or mediastinal air [1-3].

Pathophysiologically, rupture typically occurs in the distal left posterolateral esophagus; contamination with gastric contents triggers chemical injury, polymicrobial mediastinitis, cytokine surge, and endothelial dysfunction, and, if untreated, can lead to dehydration, mediastinitis, sepsis, acute respiratory distress syndrome, massive pleural effusion, esophageal fistula, empyema, shock, and death [3,4]. These mechanistic cascades explain the dramatic time-dependence of outcomes.

Contrast-enhanced CT is the investigation of choice, as it can rapidly detect mediastinal air, pleural effusions, and the site of perforation [4,5]. The “golden 24 hours” remains a pragmatic threshold, as early recognition reduces mortality, re-interventions, and length of stay.

Management should be multidisciplinary. Hemodynamically stable patients with contained perforations may be treated conservatively with nil per os (NPO), broad-spectrum intravenous antibiotics, and nutritional support. However, patients with uncontained leaks or sepsis generally require urgent surgical repair and drainage. Endoscopic stenting or vacuum therapy may be considered in selected cases [5-9].

## Conclusions

Boerhaave syndrome is a rare, life‑threatening emergency that requires a high degree of clinical suspicion, particularly in patients presenting with severe chest pain after forceful vomiting. Early recognition of surgical emphysema and rapid CT imaging are crucial for diagnosis. Timely multidisciplinary management significantly improves survival. While surgical repair is the cornerstone for most cases, conservative management with broad-spectrum intravenous antibiotics, NPO, and nutritional support may be appropriate for contained or minor perforations in stable patients. This case illustrates the importance of thorough physical examination, prompt imaging, and early transfer to a specialized center for definitive intervention.
